# Evolution of gray zone after acute myocardial infarction: Influence of microvascular obstruction

**DOI:** 10.1186/1532-429X-13-S1-P151

**Published:** 2011-02-02

**Authors:** Nilesh R Ghugre, Mohammad I Zia, Perry Radau, John J Graham, Kim Connelly, Alexander J Dick, Graham A Wright

**Affiliations:** 1 Sunnybrook Health Sciences Centre, Toronto, ON, Canada; 2 St. Michael's Hospital, Toronto, ON, Canada; 3 University of Ottawa Heart Institute, Ottawa, ON, Canada

## Introduction

The presence of ischemia-induced microvascular obstruction (MVO) despite successful coronary revascularization, has been associated with poor functional recovery and adverse left ventricular remodeling after acute myocardial infarction (AMI). Additionally, the extent of the infarct gray zone is a strong independent predictor of post-AMI mortality. However, the evolution of gray zone after AMI has not been investigated and its relationship with MVO in terms of risk stratification is unknown.

## Purpose

To characterize the evolution of gray zone and determine its correlation with the presence of MVO during infarct healing in patients treated with primary percutaneous coronary intervention (PCI).

## Methods

Patients were enrolled post-PCI and underwent MRI examination on a 1.5T scanner (GE Signa Excite) at day 2, week 4 and month 6 following AMI. Cardiac function [Ejection fraction (EF), End-diastolic volume (EDV)] was evaluated using a steady-state-free-precession (SSFP) sequence in cine mode. A T1-weighted IR-GRE sequence was used for delayed-hyperenhancement (DHE) of infarcted myocardium. Infarct core (IC) and gray zone (GZ) volumes were quantified using the full-width-half-maximum technique as previously described; both quantities were expressed as a percentage of myocardial volume. MVO’s were manually traced and included in the infarct core calculation.

## Results

**Figure 1:**
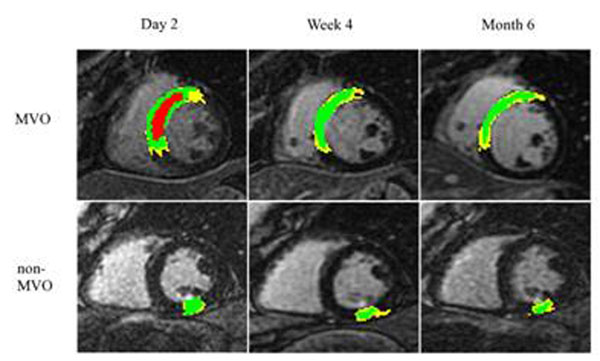
Delayed-enhanced short-axis slices from two representative patients in the MVO and non-MVO groups imaged at three different time points post AMI. Color coding identifies three regions: Infarct core (green), gray zone (yellow) and microvascular obstruction (red).

**Figure 2:**
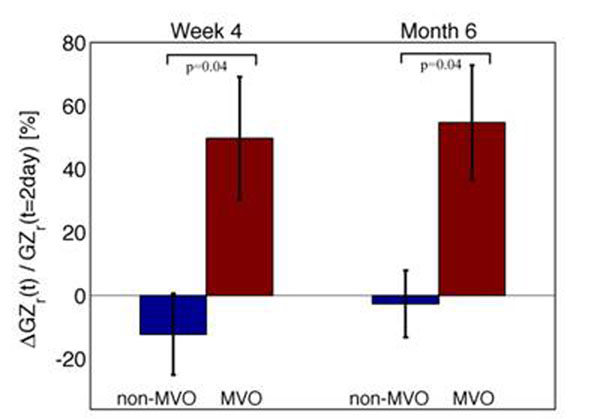
Plot demonstrates the change in GZr at two time points (t) i.e. week 4 and month 6 relative to the initial measurement at day 2. Error bars indicate standard error.

Ten patients who completed MRI exams at all three time points were included in the study [mean age: 59.7±11 years; 9 males, 1 female; 4 right coronary artery and 2 left circumflex artery PCI; MVO was identified in 4 patients]. Figure 1 shows DHE images from representative patients in the MVO and non-MVO groups while Tables 1, 2, 3 summarize the corresponding quantities measured. Note that the following findings were significant: (a) infarct core was reduced in all patients by month 6; (b) MVO was resolved by week 4; (b) gray zone size relative to infarct size, GZ_r_ = 100*GZ/(GZ+IC) increased in the MVO group at month 6 while it remained stable in the non-MVO group; (d) EF was reduced in the MVO group compared to the non-MVO at all time points (p<0.05). The percent change in GZ_r_ (ΔGZ_r_) at week 4 and month 6, relative to day 2 is shown in Figure 2.

**Table 1:** All patients (N=10)

	Day 2	Week 4	Month 6
GZ (%)	7.8±3.5	7.4±6.0	7.2±5.0
IC (%)	18.5±11.6	12.2±8.1 *	11.5±8.1 *
MVO (%)	3.5±5.1	0.2±0.3	0.2±0.4
EF (%)	47.4±7.1	49.8±7.8	51.5±11.1
EDV (ml)	123.2±30.3	143.9±33.0	117.0±23.8
GZr (%)	31.4±7.8	37.4±17.0	38.1±11.7

Values have been expressed as mean±standard deviation.

p-values were measured with respect to day 2 measurements by paired Students t-test

* p<0.01

**Table 2:** non-MVO patients (N=6)

	Day 2	Week 4	Month 6
GZ (%)	6.6±3.1	4.4±2.3 **	4.5±2.7
IC (%)	11.8±4.5	8.9±6.6	7.5±5.3 **
MVO (%)	0	0	0
EF (%)	50.8±7.3	53.3±8.3	57.5±7.1
EDV (ml)	134.6±27.4	135.0±22.9	117.6±23.7
GZr (%)	34.5±5.7	30.3±11.8	33.6±10.2

Values have been expressed as mean±standard deviation.

p-values were measured with respect to day 2 measurements by paired Students t-test

** p<0.05

**Table 3:** MVO patients (N=4)

	Day 2	Week 4	Month 6
GZ (%)	9.7±3.6	12.0±7.2	11.3±5.0
IC (%)	28.5±12.0	17.2±8.2 **	17.5±8.5 **
MVO (%)	8.7±4.2	0.4±0.5 **	0.4±0.6 **
EF (%)	42.2±3.0	44.6±3.0	42.3±10.0
EDV (ml)	106.1±29.1	157.3±44.7 *	116.2±27.6 *
GZr (%)	26.1±8.7	39.4±18.5	38.9±10.7 **

Values have been expressed as mean±standard deviation.

p-values were measured with respect to day 2 measurements by paired Students t-test

* p<0.01, ** p<0.05

## Conclusions

The increase in infarct gray zone or arrhythmogenic substrate may be one of the possible mechanisms responsible for adverse remodeling and poor clinical prognosis in patients with MVO. Monitoring the early evolution of gray zone in the high-risk patients could also potentially help identify the optimal timing and candidates for placement of implantable cardioverter-defibrillator.

